# Similar effects between animal-based and plant-based protein blend as complementary dietary protein on muscle adaptations to resistance training: findings from a randomized clinical trial

**DOI:** 10.1080/15502783.2025.2568047

**Published:** 2025-10-08

**Authors:** Martin Hindermann Santini, Alice Erwig Leitão, Bruna Caruso Mazzolani, Fabiana Infante Smaira, Mariana Silva Camargo de Souza, Andrea Santamaria, Bruno Gualano, Hamilton Roschel

**Affiliations:** a Applied Physiology and Nutrition Research Group – School of Physical Education and Sport and Faculdade de Medicina FMUSP Universidade de Sao Paulo, Sao Paulo, Brazil; b University of Sao Paulo, Center of Lifestyle Medicine, School of Medicine, Sao Paulo, Brazil; c Hospital Das Clínicas HCFMUSP, Faculdade de Medicina FMUSP, Universidade de Sao Paulo, Laboratory of Assessment and Conditioning in Rheumatology, São Paulo, Brazil

**Keywords:** Dietary intake, plant-based protein, protein source, strength, muscle hypertrophy

## Abstract

**Background:**

Recent evidence suggests that both animal and plant proteins support strength and hypertrophy gains when paired with resistance training and adequate protein intake. The purpose of this study was to investigate the effects of supplementary protein source (blend of plant-based [PLNT] vs. animal based [ANML]) to habitual diet on changes in muscle mass and strength in healthy young men undertaking resistance training.

**Methods:**

Forty-four young untrained males were recruited for this study. Subjects were randomly allocated into two groups, and consumed three 15-g daily doses (45 g. d-^1^ total) of either a mixed plant- (i.e.; soy and pea) or animal-based (i.e.; whey) protein in drink form as a supplementary source of protein in their main meals of the day (i.e.; breakfast, lunch, and dinner) for 12 weeks combined with a 3 times/week linear periodized and supervised resistance training program. Prior to the start of the trial, three (2 nonconsecutive weekdays and 1 weekend day) 24-h dietary recalls were collected to determine baseline habitual protein intake and were repeated during the protocol at weeks 4, 8, and 12. Muscle cross-sectional area (CSA, via ultrasonography imaging) determination, body composition (via dual emission x-ray absorptiometry, DXA), and lower-body maximum dynamic strength (1RM, via leg press) were assessed at baseline (PRE) and after 12 weeks of intervention (POST).

**Results:**

Both groups showed significant (all *p* < 0.0001) PRE-to-POST increases in whole-body lean mass (PLNT: 2.4 kg ±1.6, ANML: 2.5 kg ±3.9), appendicular lean mass (PLNT: 1.2 kg ±0.2; ANML: 1.8 kg ±0.2) and leg lean mass (PLNT: 0.9 kg ±0.2; ANML: 1.3 kg ±0.2), vastus lateralis mCSA (PLNT: 0.9 cm^2^ ±0.2; ANML: 1.3 cm^2^ ±0.2) and leg-press 1RM (PLNT: 64 kg ±7.8; ANML: 63 kg ±7.5), with no between-group differences for any of the variables (all *p* > 0.05).

**Conclusion:**

Complementing dietary protein intake with either a blend of plant- or an animal-based protein similarly supported resistance training-induced muscle adaptations.

**Trial registration:**

Distinct Sources of Supplementary Protein in the Resistance Exercise Training-induced Adaptations, NCT05710614, 08/01/2023.

## 1. Introduction

Plant-based proteins have become increasingly popular over recent years primarily due to their association with healthy aging and various positive health outcomes [1]. Despite current knowledge demonstrating the effects of protein on muscle anabolism to be a function of its essential amino acid (EAA) profile, there is still controversy as to whether animal-based proteins may confer any anabolic advantage over plant-based proteins.

The muscle protein synthetic response (i.e.; increase in muscle protein synthesis rates [MPS]) to protein intake depends on post-prandial availability of EAA [2], in particular leucine, which varies significantly between different protein source [3–6]. In this respect, plant- and animal-based proteins diverge in their EAA content [6,7] and digestibility [8], which seems to impact the subsequent amino acid delivery pattern [9], although other studies have shown otherwise [10,11]. In fact, a number of studies have consistently shown lower acute anabolic responses to plant (e.g. soy or wheat) than animal (e.g. whey or milk) protein, in protein-matched conditions combined [3,5,12] or not [4,5,12] with resistance exercise.

Despite the above, literature is not consistent regarding long-term effects of different supplementary protein sources, in combination with resistance exercise training (RT) program, on muscle gains, with some studies showing similar results [10,13–16] whilst others point in favor of animal protein [5,17–19]. This may be, at least partially explained, by the use of plant-based protein blends, rather than isolated, in comparison to animal-based protein [14,16,20–23]. The aim of the present study is to compare the effects of a blend of plant- (i.e.; soy and pea protein) vs. an animal-based (i.e.; whey protein) protein drink as complementary dietary proteins to habitual intake on resistance-exercise mediated gains in muscle strength and mass under an optimal protein intake condition (i.e.; ~1.6 g.kg^−1^. d^−1^) [24,25]. We hypothesized that both protein sources would be equally effective in supporting muscle anabolism in response to resistance training.

## 2. Methods

### 2.1. Experimental design

**Figure 1.**
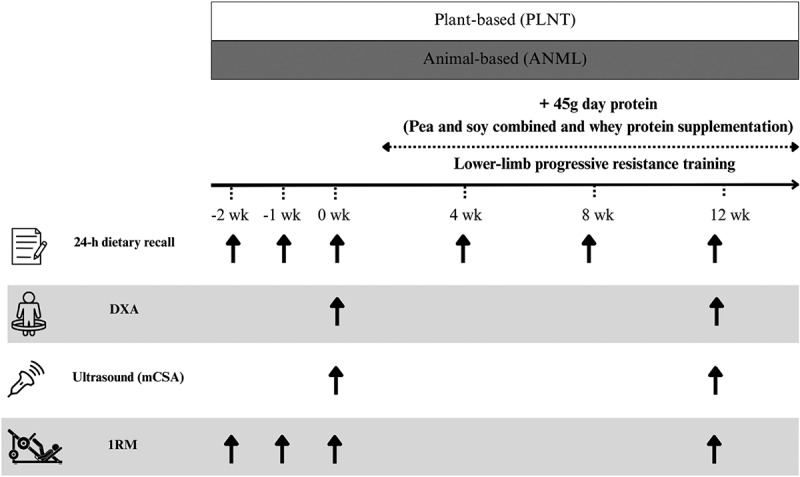
Schematic representation of the experimental design. DXA, Dual-Energy X-ray Absorptiometry; mCSA, muscle cross-sectional area; 1RM, one-repetition maximum isotonic strength.

This is a double blind randomized controlled trial to compare the effects of supplementary protein of different sources (i.e.; plant- vs animal-based protein) to habitual dietary intake on muscle adaptations to RT. Young, healthy, and recreationally active participants consuming an omnivorous diet with protein intake within RDA (i.e.; 0.8-1.0 g.kg-^1^. d-^1^) were recruited to a 3 times/week, 12-week, supervised, lower-limb focused, RT program in combination with the intake of three 15-g daily doses (45 g. d-^1^ total) of either a blend of plant- (i.e.; soy and pea) or animal-based (i.e.; whey) protein in drink form as a supplementary source of protein in their main meals of the day (i.e.; breakfast, lunch, and dinner). Before (PRE) and after (POST) the 12-week intervention, participants were assessed for body composition (dual emission x-ray absorptiometry determined body composition, DXA), muscle cross-sectional area (ultrasound imaging), and maximal lower-limb isotonic strength (maximal isotonic strength test,1RM on a leg-press). Training was carried in a laboratory setting and was individually monitored by an experienced researcher blinded to group allocation. Figure 1 illustrates the experimental design.

### 2.2. Participants

Forty-four healthy young men aged between 18-35 y were recruited to take part in the study by means of advertisements on campus and social media. Participants were randomized (1:1) into either the mixed plant- (PLNT) or animal-based (ANML) protein group using a computer-generated randomization code. Inclusion criteria were: physically active (according to the International Physical Activity Questionnaire (IPAQ) [26]; absence of any chronic condition (i.e.; type 2 diabetes, hypertension) that could preclude participation in a RT program or physical testing; habitual protein consumption within RDA ≥0.8 ≤ 1.0 g.kg^−1^. d^−1^. The exclusion criteria included prior history of anabolic steroids use, current or previous (≤3 m) use of ergogenic or protein-based supplements, current or previous ( < 1y) engagement in energy-restricted diets.

### 2.3. Dietary intake

Prior to the start of the trial, three (2 nonconsecutive weekdays and 1 weekend day) 24-h dietary recalls were collected in order to determine baseline habitual protein intake. Dietary intake throughout the trial was monitored via three additional 24-h dietary recalls at weeks 4, 8, and 12. All 24-h dietary recall interviews were collected in-person using the USDA Automated Multiple-Pass Method, a standardized validated method which uses five memory cues (1: Quick list; 2: Forgotten foods list; 3: Time and occasion; 4: Detail and review; and 5: Final probe) to elicit recall of all possible foods consumed [27]. Portion size aids were used during each interview by means of food booklets with household measures [28,29] and real-size food pictures [30].

All procedures were conducted by a trained registered dietitian, blinded to the protocol, and data were analyzed by the same trained professional using specific software (Nutritionist Pro® v.7.3, Axxya Systems, Woodinville, WO, USA). During the intervention, participants were constantly recommended to maintain their habitual dietary intake and refrain from using any other supplements that could influence training performance and/or body composition (e.g. creatine, caffeine, other protein supplements, beta-alanine, nitrate, and sodium bicarbonate).

### 2.4. Body composition

Whole-body lean mass, appendicular lean mass, leg lean mass and whole-body fat mass were assessed by DXA using Hologic QDR™ densitometry equipment (Series X-Ray Bone Densitometer, Hologic Inc. 36 Apple Ridge Road Danbury, CT 06810, USA). Scans were carried in the morning after an overnight fast at PRE and POST. Measurements were conducted by a trained investigator blinded to the protocol. The test-retest coefficient of variation for lean-mass DXA assessments was 0.4% in our laboratory.

### 2.5. Muscle cross-sectional area (mCSA)

Vastus lateralis cross-sectional area (mCSA) was assessed at PRE and POST by a B-mode ultrasound with a 7.5-MHz linear-array probe (SonoAce R3, Samsung-Medison, Gangwon-do, South Korea) as previously described [31]. MCSA analyses were performed in a blinded-fashion by a single investigator using ImageJ (NIH, USA). Test-retest typical error and coefficient of variation for vastus lateralis mCSA were 0.59 cm^2^ and 2.1%.

### 2.6. Maximal isotonic strength test (1RM)

Before testing, all participants performed two familiarization sessions separated by at least 72 h. Lower-limb strength was assessed on an incline leg-press (45°), (Nakagym®, Diadema, Sao Paulo, Brazil) following recommendations of the American Society of Exercise Physiologists [32]. Test – retest typical error and coefficient of variation for 1 repetition maximum (RM) testing were 6 kg and 2.4%. Participants performed a general warm-up on a treadmill (Movement®, Pompeia, SP, Brazil) walking for five minutes. Then, they performed a specific warm-up consisting of a set of eight repetitions with an estimated 50% 1RM followed by a set of three repetitions with an estimated 70% 1RM, two minutes apart. The 1RM test started after a 3-min rest and measured the maximum amount of weight that could be lifted in a complete movement cycle, which began and ended with the knees in full extension, reaching 90° flexion in the descendent phase of the movement. The 1RM value was determined with a maximum of five attempts with 3-min rest intervals between them [32] by an experienced research staff blinded to group assignment.

### 2.7. Protein supplementation protocol

Protein supplementation was offered thrice daily (training and non-training days), along with the main meals (individual 250-ml bottles were taken with breakfast, lunch and dinner) throughout all the 12-week experimental intervention. Plant- and animal-based protein supplements were presented in “ready to drink” format of similar color and taste (Not.co®). Each 250-ml dose contained 15 g of either a mix of soy and pea protein or whey protein. The nutritional composition of both supplements is shown in Table 1. Participants were requested to return empty bottles of the supplement to verify compliance alongside a daily log of intake registers. At the end of the follow-up, participants were surveyed about which supplement they believed to have taken. The percentage of correct answers was compared between groups to test the effectiveness of the blinding procedure.

**Table 1.** Nutritional composition of the protein supplements (per serving = 250 ml).

	Mixed Plant-based protein	Animal-based protein
Energy	110 kcal	110 kcal
Carbohydrates	6.8 g	2 g
Sugars	0.5 g	<0.5 g
Protein	15.3 g	16,2 g
EAAs	5.5 g	5 g
Leucine	1.1 g	1 g
Fat	2.5 g	4.5 g
Dietary fiber	2.0 g	2.3 g

The mixed plant-based protein beverage contains soy and pea proteins, while the animal-based version contains whey protein. Both are ready-to-drink. EAAs, essential amino acids.

### 2.8. Resistance training program

The 12-week, lower-limb focused, supervised RT program was comprised of incline leg-press 45º (Nakagym®, Diadema, Sao Paulo, Brazil), leg-extension (Nakagym®, Diadema, Sao Paulo, Brazil), and leg-curl exercises (Nakagym®, Diadema, Sao Paulo, Brazil), performed on nonconsecutive days (Monday Wednesday and Friday). All training sessions were carried during the same time of day for each participant, according to availability (either before lunch: ~11 am or before dinner: ~5pm). Training program followed a progressive overload as follows: weeks 1 to 4: 2 sets of 12-15-RM; weeks 5 to 8: 3 sets of 10-12-RM; weeks 8 to 12: 4 sets of 8-10-RM. Resistance was increased whenever the individual was able to perform one or two repetitions over the pre-established number on two consecutive sets [33]. Two minutes of rest were given between sets for all sessions. A trained member of the research team supervised all training sessions whilst remaining blinded to the protocol. A training log of each exercise session was kept for adherence’s monitoring and training volume load calculation (sets ×repetitions ×resistance for both leg press leg extension and leg-curl exercises) [34].

### 2.9. Statistical analyses

Sample size was calculated using mCSA for vastus lateralis as the primary outcome. Analyses were run using G*Power® (3.1.9.2) performing a two-way ANOVA with repeated measures (within-between interaction) considering a medium effect size (f = 0.25) and setting power to 80% (β = 0.2), with α = 0.05, which yielded an estimate of *n* = 17 per group. Due to potential dropouts, we aimed for 22 participants per group. Effects of supplementary protein source on dependent variables and between-group differences in adherence to the intervention protocol and training volume load throughout the resistance training program were analyzed using mixed-model for repeated measures assuming “group” (PLNT and ANML) and “time” (PRE and POST) as fixed factors and “subjects” as random factor. Dietary intake throughout the intervention period was assessed by mixed-model for repeated measures assuming “group” (PLNT and ANML) and “time” (PRE, weeks 4, 8, and 12) as fixed factors, and “subjects” as random factor. The association between the groups (PLNT vs. ANML) and the accuracy in identifying the beverage was assessed using Fisher’s Exact Test. Data was analyzed using the software SAS® 9.4 (SAS Institute, Inc., Cary, NC, USA), and the level of significance was set at *p* < 0.05.

## 3. Results

### 3.1. Participants

**Figure 2.**
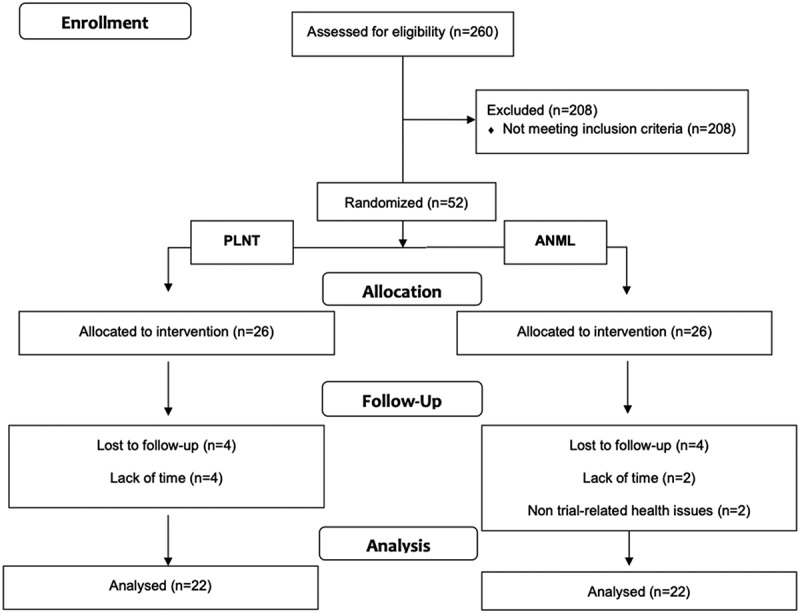
Flow chart of participants.

Figure 2 shows the flow of the participants. Two hundred and sixty participants were assessed for eligibility. Two hundred and eight did not meet the inclusion criteria. Fifty-two participants were randomized to the intervention. Four participants dropped out in the PLNT group due to personal reasons, whereas two participants dropped out due to lack of time and two due to non-trial-related issues in the ANML group, and were excluded from the analysis. Table 2 shows baseline characteristics for the participants who completed the trial (*n* = 22 PLNT group and *n* = 22 ANML group). Participants were comparable for age, body weight, height, whole-body fat mass, leg lean mass, appendicular lean mass, whole body fat mass, vastus lateralis mCSA and leg-press 1RM.

**Table 2.** Baseline characteristics of the participants.

	PLNT (*n* = 22)	ANML (*n* = 22)	*p* value
Age, y	25 (4.4)	26 (4.3)	.60
Body weight, kg	74 (8.3)	74 (8.5)	.85
Height, cm	1.77 (0.1)	1.76 (0.1)	.60
BMI, kg·m2^−1^	23.7 (2.8)	23.7 (2.5)	.95
Whole-body lean mass, kg	50.2 (4.8)	49.1 (5)	.45
Appendicular lean mass, kg	23.3 (2.6)	22.4 (2.5)	.21
Leg lean mass, kg	17.5 (1.9)	16.5 (1.8)	.10
Whole-body fat mass, kg	20.5 (5.3)	21.4 (5.8)	.58
Leg-press 1RM, kg	215 (55)	198 (40)	.23
Relative strength*, kg·kg body weight^−1^	2.9 (0.6)	2.7 (0.4)	.19
PA level, met·min·week^−1^	1433 (708)	1419 (570)	.94
Muscle CSA VL, cm2	25.3 (4.1)	24.7 (4.8)	.45

Values are presented as means (standard deviation). PLNT, plant-based dietary supplement; ANML, animal-based dietary supplement; BMI, body mass index; 1RM, maximal isotonic strength; PA, physical activity; CSA VL, cross sectional area of the vastus lateralis. *Relative strength for the leg-press 1RM test.

### 3.2. Dietary intake

Dietary intake at baseline and during the intervention is presented in Table 3. Adherence to the intervention (supplementation) was more than 90% for both groups. As per design, protein, EAAs (and leucine) intake increased over time (*p* < 0.05), with no significant group-by-time or between-group interactions (*p* > 0.05). Carbohydrate and dietary fiber intake remained stable over the intervention (*p* > 0.05). A significant main effect of group was detected for carbohydrate intake (*p* < 0.05); however, no significant group-by-time interaction was observed (*p* > 0.05). Both total energy and fat intake increased over time (*p* < 0.05), with no group-by-time interaction (*p* > 0.05).

**Table 3.** Dietary intake at baseline and during weeks 4, 8, and 12 of the intervention.

	Week 0		Week 4		Week 8		Week 12		
	PLNT	ANML	PLNT	ANML	PLNT	ANML	PLNT	ANML	p
Energy, kcal·day ^−1^ #	1881 (469)	1892 (406)	2503 (564)	2188 (438)	2183 (694)	2131 (295)	2308 (461)	2142 (267)	0.30
Protein, g·kg ^−1^ #	1 (0.2)	1 (0.2)	1.8 (0.2)	1.8 (0.4)	1.8 (0.4)	1.8 (0.3)	1.7 (0.3)	1.8 (0.2)	0.95
EAA, g·day ^−1^ #	28 (7)	28 (6)	46 (5)	51 (10)	48 (12)	53 (7)	48 (14)	52 (7)	0.42
Leucine, g·day ^−1^ #	5.2 (1.7)	5.5 (1.5)	9.1 (1.7)	9 (2.1)	9.5 (2.7)	9.1 (1.9)	9.2 (2.2)	9.1 (1.2)	0.72
CHO, g·day ^−1^ *	246 (71)	232 (70)	289 (98)	220 (81)	241 (134)	202 (38)	256 (82)	211 (55)	0.39
Fat, g·day -^1^ #	65 (27)	70 (22)	82 (27)	80 (16)	73 (23)	85 (25)	77 (27)	87 (21)	0.14
Dietary fiber, g·day^−1^	21 (9)	20 (8)	23 (11)	21 (12)	22 (9)	19 (7)	20 (9)	23 (10)	0.40

Values are presented as means (standard deviation). PLNT, plant-based dietary supplement; ANML, animal-based dietary supplement; CHO, carbohydrates; EAA, essential amino acids; Week 0 represents baseline nutritional intake before the intervention. Weeks 4, 8, and 12 include the 45 g of supplementary protein. Dietary data were analyzed with mixed-model for repeated measures. #Indicates *p* < 0.05 for main effect of time (when compared to week 0), * Indicates *p* < 0.05 for main effect of group. *p*, group by time interaction.

### 3.3. Body composition

**Figure 3.**
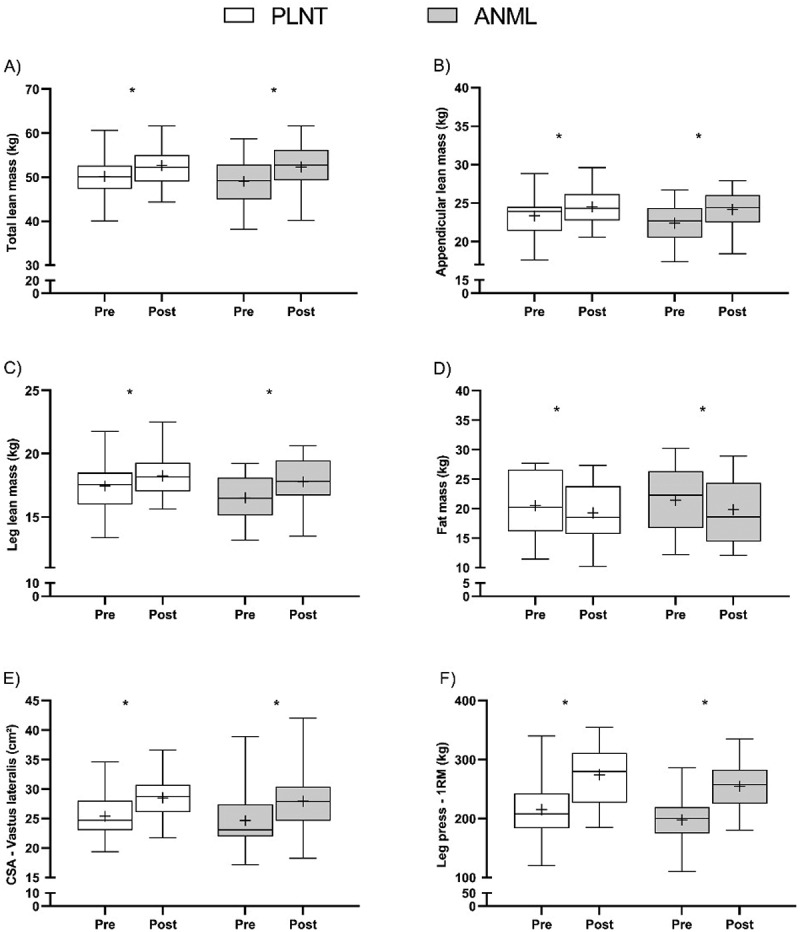
Panel A: total lean mass (kg); Panel B: appendicular lean mass (kg; Panel C: leg lean mass (kg); Panel D: fat mass (kg); Panel E: mCSA - vastus lateralis muscle cross sectional area (cm^2^); Panel F: leg press - 1RM (kg). Values are presented as median (lines) with interquartile range (boxes), minimum and maximum (whiskers), and mean (+). PRE: before and POST: after the intervention PLNT exclusive plant-based complementary protein consumers, ANML animal-based complementary protein consumers, CSA cross sectional area. *Indicates significantly different from PRE (p < 0.05 for the main effect of time).

We observed a main effect of time for whole-body lean mass, appendicular lean mass and leg lean mass (all *p* < 0.01) with no group-by-time interaction (*p* > 0.05). Whole-body lean mass increase from 50.21 ± 4.78 kg to 52.62 ± 4.37 kg (*p* < 0.01) in PLNT, and from 49.08 ± 4.97 kg to 52.31 ± 5.19 kg (*p* < 0.01) in ANML; whereas appendicular lean mass increased from 23.33 ± 2.55 kg to 24.52 ± 2.23 kg (*p* < 0.01) in PLNT, and from 22.37 ± 2.48 kg to 24.16 ± 2.49 kg (*p* < 0.01) in ANML (Figure 3, Panels A and B). A similar pattern was observed for leg lean mass. We detected a main effect of time (*p* < 0.01) with no group-by-time interaction (*p* > 0.05). PLNT increased leg lean mass from 17.38 ± 1.87 kg to 18.26 ± 1.65 kg (*p* < 0.01), whereas ANML increased from 16.52 ± 1.75 kg to 17.80 ± 1.86 kg (*p* < 0.01) (Figure 3, Panel C).

Whole-body fat mass decreased regardless of group (main effect of time, *p* < 0.05; with no group-by-time interaction, *p* > 0.05). PLNT decreased whole-body fat mass from 20.49 ± 5.32 kg to 19.27 ± 5.14 kg (*p* < 0.05), whereas ANML decreased from 21.42 ± 5.77 kg to 20.19 ± 5.48 kg (*p* < 0.05) (Figure 3, Panel D).

### 3.4. Muscle cross sectional area

There was a main effect of time for increase in vastus lateralis mCSA (*p* < 0.01) with no group-by-time interactions (*p* > 0.05). PLNT vastus lateralis mCSA increased from 25.36 ± 4.14 cm^2^ to 28.53 ± 3.64 cm^2^, whilst ANML increased from 24.65 ± 4.80 cm^2^ to 27.90 ± 5.47 cm^2^ (both *p* < 0.05 for within-group comparisons) (Figure 3, Panel E).

### 3.5. Maximal isotonic strength

There was a main effect of time for leg-press 1RM (*p* < 0.01) with no group-by-time interaction (*p* > 0.05). PLNT increased 1RM from 215 ± 55 kg to 279 ± 55 kg, whereas ANML increased from 197 ± 39 kg to 255 ± 42 kg (both *p* < 0.01 for within-group comparisons) (Figure 3, Panel F).

### 3.6. Adherence, adverse effects, blinding and training volume load

PLNT and ANML completed 80 ± 9% and 84 ± 6% of all prescribed training sessions, respectively (*p* > 0.05). Adherence to supplementation was very high and similar across groups (*p* > 0.05): PLNT = 93 ± 9%; ANML = 96 ± 6%. The blinding procedure was effective, with no significant difference between PLNT (45% correct answers) and ANML (57% correct answers) (*p* > 0.05). No adverse effects of either training or supplementation were reported throughout the trial.

Training volume load was similar between groups. Leg press volume load was 285 ± 50 10^3^ kg and 268 ± 68 10^3^ kg for PLNT and ANML, respectively; leg extension and leg curl volume load were 120 ± 20 10^3^ kg and, 119 ± 19 10^3^ kg; and 120 ± 15 10^3^ kg and 117 ± 18 10^3^ kg for PLNT and ANML, respectively (all *p* > 0.05).

## 4. Discussion

In the present study, we compared the effects of a blend of plant- (soy and pea protein) vs. animal-based (whey protein) protein drinks as complementary dietary proteins to habitual intake on muscle adaptations in response to a 12-week RT program. Our findings revealed no significant differences in RT-induced increases in whole-body, appendicular and leg lean mass, muscle cross-sectional area, and muscle strength between the two groups, regardless of the supplementation protein sources.

Literature have consistently shown the effects of dietary protein on MPS stimulation [35]. In this respect, the muscle protein synthetic response to protein intake seems to rely on the post-prandial availability of EAA [25,36], particularly on leucine [2], which exhibits significant variability among different protein sources [6,19]. Indeed, several studies have compared MPS in response to different protein sources, either at rest or following exercise [3,4,12,37]. The majority of these studies have observed superior MPS after the ingestion of animal- compared to plant-based proteins [3,5,12]. However, results from studies assessing acute effects of MPS do not directly translate into long-term increases in muscle mass or strength, which are chronic outcomes associated with exercise and protein intake [35].

Our results show similar effects of either complementary protein source on increases in muscle mass and strength in response to a structured RT program. These results are aligned with our recent study [16], replicated by an independent group [14], reporting comparable effects of vegan vs. omnivorous high-protein diets on muscle adaptations to RT. Despite differences in EAA composition between protein sources in the present and previous [14,16] studies, at least two factors help explaining the results.

Firstly, leucine is considered the main trigger for protein-mediated increase in MPS [36,38]. Additionally, the effects of leucine on muscle anabolism are both dose-dependent [39] and saturable [40]. Indeed, leucine levels were comparable between PLNT and ANML groups in the present study, thus supporting the notion of similar anabolic potential across groups. Secondly, reduced digestibility of an isolated plant-, compared to animal-based protein, in association with an allegedly higher-quality EAA profile in the latter, may limiting plant-based protein-derived EAA availability to skeletal muscle [41]. In this respect, combining different plant-derived protein blends may provide a more balanced amino acid profile. Pinckaers et al. [20] have demonstrated that a plant-based protein blend (wheat/corn/pea protein) induced similar increases in MPS than animal-based protein (whey), regardless of different EAA concentrations, indicating a ceiling-effect of protein/EAA/leucine on MPS. Askow et al [42] have recently shown that a 9-day vegan eating pattern associated with RT resulted in similar stimulation of MPS rates in comparison to a protein-matched (~1.2 g.kg^−1^. d^−1^) omnivorous diet, further supporting the notion of the beneficial effects of plant-based protein diversity in comparison to single source or isolated plant proteins. Finally, we [16] and others [14] have shown that mixed plant-based diets providing adequate protein amounts (~1.6 g.kg^−1^. d^−1^) are similarly effective in supporting RT-mediated increases in muscle mass and strength than protein-matched omnivorous diets.

To the best of our knowledge, this is the first trial to evaluate the effects of a mixed plant-based (blend of soy and pea) in comparison to animal-based protein supplementation on muscle adaptations to RT. Our findings contribute to the growing body of evidence regarding the effects of different protein sources on long-term effects on muscle adaptations to exercise. It is important to acknowledge that these findings are derived from a context of an omnivorous diet, which may limit the generalizability of the results to plant-based or animal-free diets. The present study has some limitations that should be considered when interpreting the results. Our sample consisted of young, healthy, recreationally active men following an omnivorous diet, which may limit the generalizability of the findings to other populations with different characteristics or dietary patterns, including female population. Protein supplementation dose was not individualized across participants, hence, it may not represent individualized, or optimal protein, intake patterns for all individuals. The focus on lower-limb muscle assessments may not fully capture muscle adaptations in other body regions. Our sample was composed of nonresistance-trained individuals, which may adapt more rapidly to training, thus making possible differences between interventions. Finally, our study design did not incorporate a non-protein placebo group, which limits inferences on the effectiveness of increasing protein – of either source – on muscle adaptations. Future studies should address these limitations to provide a more comprehensive understanding of different sources of protein supplementation and its effects on muscle adaptations in diverse populations and contexts.

In conclusion, our findings revealed that complementing dietary protein intake with either a plant-based blend or animal-based proteins in drinking form similarly support RT-mediated increase in muscle mass and strength in health, young, physically-active individuals under optimal protein intake.
